# Role of the Inflammation-Autophagy-Senescence Integrative Network in Osteoarthritis

**DOI:** 10.3389/fphys.2018.00706

**Published:** 2018-06-25

**Authors:** Claire Vinatier, Eduardo Domínguez, Jerome Guicheux, Beatriz Caramés

**Affiliations:** ^1^INSERM, UMR 1229, Regenerative Medicine and Skeleton, University of Nantes, ONIRIS, Nantes, France; ^2^University of Nantes, UFR Odontologie, Nantes, France; ^3^Biofarma Research Group, Center for Research in Molecular Medicine and Chronic Diseases, University of Santiago de Compostela, Santiago de Compostela, Spain; ^4^CHU Nantes, PHU4 OTONN, Nantes, France; ^5^Grupo de Biología del Cartílago, Servicio de Reumatología. Instituto de Investigación Biomédica de A Coruña, Complexo Hospitalario Universitario de A Coruña, Sergas, A Coruña, Spain

**Keywords:** inflammation, aging, senescence, autophagy, chondrocytes, cartilage, OA, therapeutics

## Abstract

Osteoarthritis is the most common musculoskeletal disease causing chronic disability in adults. Studying cartilage aging, chondrocyte senescence, inflammation, and autophagy mechanisms have identified promising targets and pathways with clinical translatability potential. In this review, we highlight the most recent mechanistic and therapeutic preclinical models of aging with particular relevance in the context of articular cartilage and OA. Evidence supporting the role of metabolism, nuclear receptors and transcription factors, cell senescence, and circadian rhythms in the development of musculoskeletal system degeneration assure further translational efforts. This information might be useful not only to propose hypothesis and advanced models to study the molecular mechanisms underlying joint degeneration, but also to translate our knowledge into novel disease-modifying therapies for OA.

## Introduction

Osteoarthritis (OA) is one of the most prevalent joint diseases and a leading cause of disability worldwide (GBD 2016 DALYs HALE Collaborators, 2017). Our understanding of the molecular mechanisms underlying OA, which is now considered an inflammation-associated multifactorial disorder, progressed substantially. However, no effective disease-modifying therapy is available yet and current pharmacological interventions only address pain and inflammation (Martel-Pelletier et al., 2016). Although the relationship between aging and the development of OA is not completely understood, it is becoming apparent that aging-related changes in the musculoskeletal system, in conjunction with mechanical injury and genetic factors contribute to OA (Loeser, 2009; Taniguchi et al., 2009). This novel vision of OA pathogenesis has led the OARSI consortium to re-evaluate the clinical definition to more accurately reflect the contribution of the underlying molecular mechanisms (Kraus et al., 2015).

The process of cellular senescence contributes to age-related dysfunction and chronic inflammation. Senescence essentially refers to the irreversible growth arrest that occurs when cells experience stress insults. Articular cartilage undergoes aging-associated changes in structure and biochemical composition which compromise biomechanical function and contribute to the formation of structural defects (Rahmati et al., 2017). Changes in cartilage aging include a reduction in chondrocyte density, proliferation and abnormal biosynthetic function (Loeser, 2009) as a response to extracellular stimuli in human (McAlinden et al., 2001) and mouse (Sandy and Plaas, 1986) articular chondrocytes. Senescent cells secrete proinflammatory cytokines, chemokines, and proteases, termed the senescence-associated secretory phenotype (SASP) (Kuilman et al., 2010; Soto-Gamez and Demaria, 2017). Through the inflammatory, growth-promoting, and remodeling factors that are secreted, this mechanism can explain how senescent cells alter tissue microenvironments, attract immune cells, and induce malignant phenotypes in neighboring cells (Acosta et al., 2013). Proteins that are associated with the SASP, such as IL-1β, TNF-α, IL-6, or matrix metalloproteinases (MMPs) increase with aging (Freund et al., 2010), and participate in the inflammation process. SASP is thus seen as a driver of age-related inflammation, at least under certain conditions (Rodier and Campisi, 2011). In line with these, the selective elimination of senescent cells or their deleterious effects is proposed as an intervention to reduce age-related chronic inflammation (Baar et al., 2017), enhance health span (Baker et al., 2011), and disrupt the link between aging and chronic musculoskeletal disease (Garcia-Prat et al., 2016).

Aging, which is a complex physiological process consisting of progressive decline of certain functions, coordination, loss of homeostasis, and physiological integrity (Lopez-Otin et al., 2013), is one of the major risk factors for OA. Like aging, OA is commonly described as the result of disruption of cartilaginous tissue homeostasis, where catabolic products such as damage-related molecular models (DAMP) accumulate in the joint and cause oxidative stress and inflammation (Haseeb and Haqqi, 2013). Articular chondrocytes rely on autophagy as the primary mechanism for maintaining normal function and survival (Terman et al., 2010). However, during aging, autophagy gradually decreases in chondrocytes thus inducing senescence, which ultimately results in increased OA severity (Carames et al., 2010).

Considering the growing body of evidence related to the role of inflammation-autophagy network in OA-associated chondrocyte senescence, this review describes the molecular pathways underlying age-associated changes in chondrocyte senescence that could lead to novel transformative therapies.

## Senescence in OA

### General context

Cellular senescence also called replicative senescence is a stress response characterized by a persistent cell-cycle arrest. Two different observations have led to the discovery of senescence in the 1960s: Normal non-transformed cells have a limited number of division in culture and this maximal number of cell division reached by normal human cell in culture decrease with the age of the donor (Hayflick, 1965).

Cellular senescence is concomitantly considered as beneficial or detrimental for cells (Campisi and d'Adda di Fagagna, 2007). On the one hand, cellular senescence is considered beneficial notably through its property to suppress the development of cancer, its role as a pivotal mechanism during embryonic development (Munoz-Espin et al., 2013; Storer et al., 2013) or its role in wound healing at site of tissue injury (Demaria et al., 2014). On the other hand, senescence exerts detrimental actions through its promoting effect in aging and age-related diseases as evidenced by the accumulation of senescent cells in degenerated tissues (Baker et al., 2008; Campisi, 2014) and the exponential increase in the expression of some senescent markers thereby suggesting that senescent cells contribute to aging and shorten health span (Baker et al., 2016). Even if the underlying mechanisms of cellular senescence are not fully identified, they have been associated with telomere erosion, DNA damage, oxidative stress, and inflammation and led to the description of different types of senescence. The first one is telomere-initiated cellular senescence also called DNA Damage Response (DDR). The signaling pathway activated in DDR involves the stabilization of p53 after its phosphorylation by the ataxia telangiectasia mutated kinase (ATM) (d'Adda di Fagagna et al., 2003; Deng et al., 2008). The second type of senescence is stress-induced senescence also called premature senescence, which is considered a DDR-independent pathway. This premature senescence is consecutive to a variety of stress that remains poorly understood. The third one is oncogene-induced senescence which is due to the overexpression of several oncogenes such as RAS type family (Di Mitri and Alimonti, 2016).

#### Senescence features

Senescent cells in culture undergo morphological alterations, such as increased cell size, flattening, vacuolization, and accumulation of stress granules (Kuilman et al., 2010; Rodier and Campisi, 2011). These senescent cells also exhibit specific hallmarks including (i) growth arrest, (ii) cell death resistance, and (iii) an altered gene expression leading to the production of bioactive SASP.

**(i) The growth arrest**, which is a replication failure due to the expression of cell cycle inhibitors, occurs in senescent cells following various stimuli. These stimuli can consist in DNA damage, telomere shortening, oncogene activation for the most widely described, but other stimuli such as inflammation, reactive oxygen species (ROS), mitotic stress or unresolved unfolded protein response can also drive cell growth arrest. Following to these stimuli, two main signaling pathways are often involved, the DDR-dependent pathway P53-P21^CIP1^-retinoblastoma protein (pRB) and the DDR-independent pathway P16^INK4A^-pRB. These pathways include cyclin-dependent kinase inhibitors (CDKIs) such as P21^CIP1^ (CDKN1A) or P16^INK4A^ (CDKN2A), which respectively inhibited cyclin-dependent kinase-2 (CDK2), CDK4, and CDK6. Through CDK4 and CDK6 inhibition, the p16^INK4A^ conserves pRB in their repressive forms, thus preventing G1/S phase cell cycle progression (Gil and Peters, 2006). The p53-p21 pathway inhibits CDK2 that also maintains the repressive forms of pRB, but induced G1 or G2 phase cell cycle halt (Gil and Peters, 2006). While both DDR-dependent and DDR-independent pathways are intimately linked with various common upstream regulators and downstream effectors, p16^INK4A^/pRB pathway mainly drives an irreversible growth arrest whereas the p53/p21–dependent cell growth arrest may not be irreversible (Sharpless and Sherr, 2015).

**(ii) Cell death resistance** is also a common feature of senescent cells. Indeed, senescent human fibroblasts resist apoptosis induced by growth factor deprivation or oxidative stress (Hampel et al., 2005). Senescent cells also exhibit a noticeable resistance to mitochondria-mediated apoptosis in part by the maintenance or up-regulation of the anti-apoptotic protein B cell lymphoma 2 (BCL-2) family members (Ryu et al., 2007), known to inhibit the mitochondrial outer membrane permeabilization (MOMP) and the subsequent release of pro-apoptotic cytochrome C. This BCL-2-mediated resistance to apoptosis of senescent cells has been exploited by developing senolytic compounds such as Navitoclax or ABT-737 that target BCL-2 family members (Chang et al., 2016; Yosef et al., 2016; Zhu et al., 2016). In addition, senescent cells also resist to apoptosis through the interception of Fas ligand (FasL) by decoy receptor 2 (DCR2), which is overexpressed in senescent cells. Apoptosis of senescent cells may also be stopped through the P53-mediated up-regulation of p21 which directly inhibits caspase 3 activity (Tang et al., 2006).

**(iii) The Senescence-Associated Secretory Phenotype (SASP)** is a bioactive secretome produced by senescent cells which remain metabolically active despite they are not proliferative (Coppe et al., 2010a). SASP is associated with the production of proinflammatory cytokines (IL-6, IL-1α/β), chemokines [Monocyte chemotactic protein (MCP-1/CCL2)], IL-8/CXCL8, growth regulating oncogene (GROα), growth factors [TGF-β, VEGF, insulin-like growth factor (IGF)-binding protein 7 (IGFBP7) and amphiregulin] and proteases [Matrix Metalloproteases (MMP-3, TIMP-1)] that may have potent beneficial or deleterious effects on neighboring cells and the surrounding tissues (Soto-Gamez and Demaria, 2017). SASP, through the involvement of paracrine mediators, indeed propagates senescence from cell to cell thereby exacerbating the pro-aging effects of senescent cells. Among the SASP components, IGFBP-7, plasmin activator inhibitor (PAI-1), IL-6, CXCR2 ligands (IL-8 and GROα) are known to strengthen senescence either by autocrine (IL-6) (Kuilman et al., 2008) or paracrine (IGFBP7), effects (Wajapeyee et al., 2008; Acosta et al., 2013). The transient exposure to SASP is also conversely known to promote tissue regeneration or repair by the release of growth factors or proteases (Krizhanovsky et al., 2008; Demaria et al., 2014). Whereas persistent SASP signaling can result in deleterious effects such as chronic inflammation, SASP is also involved in the immune surveillance process and elimination of senescent cells by the immune system (van Deursen, 2014).

Whatever the senescence inducers (stress-induced, oncogene-induced…) and the associated signaling pathways, all SASP regulators converge to the nuclear factor-κB (NF-κB) signaling (Chien et al., 2011). This NF-κB signaling pathway is regulated by mechanistic target of rapamycin (mTOR)-dependent protein translation (Herranz et al., 2015; Laberge et al., 2015), and the cell surface-bound IL-1α (Orjalo et al., 2009). Others signaling pathways that converge to NF-κB activation are p38 mitogen-activated protein kinase (MAPK) (Freund et al., 2011), GATA4 which is stabilized by a defective p62-mediated autophagy (Kang C. et al., 2015), the phosphoinositide 3 kinase (PI3K) (Kortlever et al., 2006) pathways and the RIG-1/IRF pathways (Liu et al., 2011). NF-κb activation and the subsequent SASP production might also be initiated by oxidative stress and increased ROS (Chandrasekaran et al., 2017).

Moreover, advanced glycation end product (AGEs) as well as HMGB1 through their interaction with the Receptor for advanced glycation end product (RAGE) (Orlova et al., 2007) also activate NF-κβ pathway. Once translocated in the nucleus, NF-κB binds to the promoter region of RAGE and enhances RAGE mRNA translation (Li and Schmidt, 1997).

#### Identification of senescent cells

Owing to the lack of a specific “magic” marker to characterize or evaluate senescence *in vivo*, senescent cells have to be identified using an association of multiple markers including SAβ-gal or lipofuscin staining (Evangelou et al., 2017), the upregulated expression of p16^INK4A^, p21^CIP1^ or p19^ARF^ and SASP (Coppe et al., 2008, 2010b). In addition to the presence of these partly selective markers, the decrease or loss of some other markers may also be instrumental to identify senescent cells. Among these markers, Nuclear High mobility group box 1 (HMGB1), which is localized in the nucleus in non-senescent cells, is secreted by senescent cells, making the loss of nuclear staining for HMGB1 a sign of senescence (Davalos et al., 2013). Similarly, the decreased expression of lamin B1 is also often used to address the presence of senescent cells (Freund et al., 2012). Below are the major markers classically used to identify senescent cells.

##### Senescence associated β-galactosidase

Senescent cells exhibit increased levels of the lysosome enzyme acidic β-galactosidase called senescent-associated-β-galactosidase (SAβ-gal). Whereas, the normal β-galactosidase activity is detectable at pH 4.5, the SAβ-gal is only detectable at pH = 6 on fresh sample with the X-gal substrate (Itahana et al., 2007; Debacq-Chainiaux et al., 2009). Due to its easy detection, SAβ-gal is the most commonly used senescence marker. However, SAβ-gal has been also detected in immortalized cells, tumor cell lines, and even in normal cells when cultured in conditions known to improve lysosome activity (Young et al., 2009). The expression of SAβ-gal is now considered not specific to senescent cells (Itahana et al., 2007; Muller, 2009) and must be combined to other markers (see below) to properly identify such cells.

##### Lipofuscin

Recently Lipofuscin, a non-degradable aggregate of oxidized lipids, proteins, oligosaccharides and metals which accumulates in the lysosomes, has been recently shown to amass with age in various post-mitotic tissues (Terman and Brunk, 2004b). It has been demonstrated that lipofuscin accumulation in senescent cells is a consequence of the senescent process (Jung et al., 2007). Georgakopoulou et al. have thus shown, through Sudan Black B (SSB) staining, that lipofuscin accumulates and co-localizes with SAβ-gal in senescent cells (Georgakopoulou et al., 2013). Interestingly, SBB staining of lipofuscin can also be achieved in paraffin-embedded materials allowing retrospective senescence analysis in archival samples.

##### Telomeres length

Telomeres are tandem TTAGGG repeated sequences located at the ends of chromosome and associated with a protein complex named “shelterin” which cape the telomere end (de Lange, 2005). The shelterin complex is composed of six-subunit comprising TRF1, TRF2, POT1, TPP1, TIN2, and Rap. This protein complex is specifically associated with mammalian telomeres and allows cells to distinguish the natural ends of chromosomes from DNA damage sites. During aging or replication theses telomeres progressively shortens in somatic cells lacking the enzyme telomerase. With this shortening, the shelterin complex is displaced and expose the telomere end that become recognized by the DNA repair machinery as a double-strand DNA break and triggers a DNA Damage Response leading to senescence (Sfeir and de Lange, 2012). Moreover, cells lacking shelterin components, such as POT1 or TRF2, exhibit a DNA damage response and premature induction of senescence (Denchi and de Lange, 2007). Telomere shortening is also increased by oxidative stress (Ludlow et al., 2014) leading to senescence and accelerating aging (Aubert and Lansdorp, 2008). Inflammation through activity of NF-κB and the pro-inflammatory cytokines contribute to telomere attrition and the onset of senescence (Zhang et al., 2016).

##### Sirtuin (SIRT)

Sirtuins are members of a family of evolutionarily conserved enzymes with NAD+-dependent deacetylase/deacylase activity. Sirtuins are involved in various biological role including cellular metabolism, genome stability and lifespan regulation (for review see Houtkooper et al., 2012). Among the seven sirtuin proteins (SIRT1–7) identified in mammals, SIRT1 is the most extensively studied. SIRT1 activation is known to prevent senescence by promoting P53 degradation (Solomon et al., 2006), whereas its inactivation increases the transcriptional activity of p53. These findings indicate that the SIRT1-p53 pathway is critical for regulating cellular senescence (Yu et al., 2017). SIRT 1 level has been shown to protect from stress induce premature senescence as well as replicative senescence (Ghosh and Zhou, 2015). SIRT 6 expression ameliorates lifespan in mice whereas its knock down induces premature death within a month (Mostoslavsky et al., 2006). SIRT6 has been shown to promote DNA damage repair process (Mao et al., 2011) and protect cells from telomere shortening (Michishita et al., 2008). SIRT6 deacetylates H3K18 to prevent mitotic errors and suppress senescence (Tasselli et al., 2017). SIRT6 also attenuates NF-κβ signaling by deacetylating histone H3 at K9 on the promoters of NF-κB target genes (Kawahara et al., 2009) suggesting that SIRT6 could regulate the SASP. As a whole, sirtuins seems to have a potential key role in protecting cells from premature senescence and accelerated aging.

##### High mobility group box-1 (HMGB1)

HMGB-1 is a non-histone nuclear protein which sustains chromosome structure and stability. Under stress conditions, HMGB1 translocates from the nucleus to the cytosol and is then released into the extracellular space to function as an alarmin, a signal of tissue and cell damage (Ugrinova and Pasheva, 2017). In senescent cells, following activation of p53 HMGB-1 is secreted in the extracellular space (Davalos et al., 2013). Released HMGB-1 then stimulates NF-κβ transcriptional activity and stimulates SASP through its fixation on various receptors such as the receptor for advanced glycation end product or toll-like receptor 4. On the contrary nuclear HMGB-1 inhibits NF-κβ activity and the subsequent SASP (Davalos et al., 2013). Therefore, the loss of HMGB1 nuclear staining is an investigated marker in senescence characterization.

##### Caveolin-1 (CAV1)

CAV1 is a major component of the caveolae structure and a trans-membrane protein well-known to regulate numerous cellular processes such as cell growth and differentiation or endocytosis but also the stress-induced senescence (Zou et al., 2011; Nguyen and Cho, 2017). CAV1 is notably upregulated during replicative and premature senescence. CAV1 mediated signaling seems to be implicated in the promoting effect of oxidative stress on cell senescence, and overexpression of CAV1 induces senescence in age-related diseases (Galbiati et al., 2001; Bartholomew et al., 2009; Volonte et al., 2015). Paradoxically, CAV1 deficient mice exhibit aging related phenotype in various organs, such as neurodegeneration, fat atrophy, and premature aging (Head et al., 2010; Briand et al., 2011). Furthermore, CAV1 deficiency has recently been shown to also induce premature senescence via mitochondrial dysfunction and SIRT-1 inactivation (Yu et al., 2017).

##### P16^*INK*4*A*^

The selective inhibitor of cyclin-dependent kinases CDK4 and CDK6, p16^INK4A^ is the second most widely used senescent marker after SAβ-gal. The expression of p16^INK4A^ is increased in senescent cells and participates in the cell cycle arrest (Alcorta et al., 1996). Aging organisms accumulate senescent cells expressing p16^INK4A^ in various tissues (Krishnamurthy et al., 2004). This accumulation contributes to the onset of age-related disorders by compromising tissue function through the loss of tissue structural integrity and depletion of tissue-specific stem cell pools (Sharpless, 2004; Martinez-Zamudio et al., 2017a). Despite its well-defined role in senescence, the use of p16^INK4A^ as a marker in mice has been hampered by the lack of specific and robust antibodies available for immunohistochemistry. The involvement of p16^INK4A^ in aging and age-related diseases was however evidenced through the use of specific transgenic mice such as INK-ATTAC (Baker et al., 2011) and p16-3MR mice (Demaria et al., 2014). The INK-ATTAC mice exhibit a FK506-binding-protein–caspase 8-enhanced GFP (FKBP–Casp8-eGFP) fusion protein under the control of a p16^INK4A^ promotor. These mice allow the detection and sorting of p16^INK4A^ positive cells as well as their elimination by activation of caspase-8 after addition of AP20187 (Baker et al., 2011). The P16-3MR mice exhibit a trimodality reporter fusion protein, which contains functional domains of a synthetic Renilla luciferase (LUC), monomeric red fluorescent protein (mRFP), and truncated herpes simplex virus 1 (HSV-1) thymidine kinase under the control of p16^INK4A^ promoter allowing to concomitantly trace and kill p16^INK4A^ expressing cells through administration of ganciclovir (Demaria et al., 2014). Of particular interest, these two models have greatly contributed to address the protective role of depleting p16^INK4A^ expressing-senescent cells in aging and age-related diseases (Baker et al., 2011).

##### Nuclear foci: senescence associated heterochromatin foci (SAHF) and DNA scar

During senescence onset in the cells, a drastic chromatin remodeling arises in the nucleus leading to the formation of DNA dense heterochromatic regions called **senescence-associated heterochromatin foci (SAHF)** (Narita et al., 2003). SAHF can be determined by visualizing the reorganization of DAPI, as well as by immunoprecipitation of heterochromatin-associated histone H3K9Me2 and H3K9Me3 and its binding partner heterochromatin protein-1γ (HP-1γ) (Zhang et al., 2007). SAHF contain at least two other proteins, namely the histone variant MacroH2A and HMGA proteins (Narita et al., 2003; Zhang et al., 2007). Whereas HMGA proteins contribute to senescence-associated proliferation arrest (Funayama et al., 2006), MacroH2A protein confers to the chromatin a resistance to ATP-dependent remodeling and binding of transcription factors that in turn contribute to cell cycle exit (Zhang et al., 2005). The persistent DDR signaling triggered following DNA double-strand breaks also leads to the formation of nuclear foci, termed **DNA segments with chromatin alterations reinforcing senescence (DNA-SCARS)** where DDR proteins accumulate allowing the DNA-SCARS detection. These foci are detected through the staining of γH2AX, the phosphorylated form of histone H2AX. Following DNA damage, Histone H2AX is phosphorylated by the ATM/ATR protein and generates bright foci (Rogakou et al., 1998). The γH2AX thereafter recruits additional ATM/ATR complexes through involvement of two other proteins, the mediator of DNA damage checkpoint (MDC1) and p53-binding protein 1 (p53BP1). These two last proteins may also be used for the detection of DNA-SCARS (Martinez-Zamudio et al., 2017b). Recently new type of DNA damage consisting in cytosolic chromatin fragments that stands out from the nucleus of cells during senescence (Dou et al., 2017). This cytoplasmic chromatin fragment is recognized as double–strand DNA by cyclic GMP-AMP synthase (CGAS) a well-known pattern recognition receptor for foreign DNA in the cytosol (Stetson and Medzhitov, 2006). Once activated, CGAS activates the stimulator of interferon genes (STING) thereby instigating the SASP production (Gluck et al., 2017).

##### The senescence-associated secretory phenotype (SASP) components

Even if the SASP components greatly vary as a function of cell type, some of them including the pro-inflammatory cytokines IL-6 and IL-8 appear highly conserved. IL-6 and IL-8 are thus considered as SASP-dependent senescence markers. In addition to IL-6 and IL8, other SASP components such as MMP-1 and−3, growth factors (GM-CSF, G-CSF, IGF), cytokines (IL-1α/β, TNF-α), and chemokines (MCP-1, MIP-1α, GROα/β) can be directly assayed in cell supernatants using Elisa or multiplex immunoassay.

### Relevance of senescence in OA and articular cartilage aging

Aging organisms accumulate senescent cells expressing p16^INK4A^ in various tissues. This accumulation contributes to the onset of age-related disorders and morbidity by compromising tissue function and structural integrity (Martinez-Zamudio et al., 2017a). During aging and OA, chondrocytes exhibit many features of senescence, including growth arrest, presence of DDR and SASP production (Price et al., 2002). Indeed, chondrocytes like many other cell types and according to the Hayflick limit, can only undergo a restricted number of cell division (Evans and Georgescu, 1983). Consistently, aging chondrocytes exhibit a decreased proliferation capacity and extracellular matrix (ECM) synthesis (Guerne et al., 1995). With age articular cartilage undergo modification such as localized fibrillation and a decreased ability to respond to various anabolic stimuli, which weakens cartilage repair (Buckwalter and Mankin, 1998). Senescent cells have been observed near the OA lesion. This accumulation of senescent cells may predispose joints to OA development (Kozhemyakina et al., 2015). In addition to age-related senescence, a stress-induced senescence may be induced by pro-inflammatory cytokines on chondrocytes. The specific hallmarks of OA-associated chondrocyte senescence are discussed below.

#### Oxidative stress

Oxidative stress increases either when ROS production is improved or when antioxidants level is decreased, and intriguingly both phenomenon occur during aging (Finkel and Holbrook, 2000). Besides, elevated ROS levels have been shown to correlate with a decreased autophagy thereby suggesting that oxidative stress contributes to tissue homeostasis rupture and OA severity (Hui et al., 2016). This increased oxidative stress is also believed to result from a mitochondrial dysfunction, since OA chondrocytes exhibit reduced mitochondrial DNA content and reduced mitochondrial mass (Wang et al., 2015). This mitochondrial dysfunction is also accompanied by a decreased expression of sirtuin 1 (SIRT1) and peroxisome proliferator–activated receptor γ coactivator 1α (PGC-1α) both involved in mitochondrial biogenesis, and a down-regulation of the nuclear respiratory factors 1 and 2 (NRF1 and NRF2), which regulate antioxidant gene expression (Wang et al., 2015). This mitochondrial dysfunction was further confirmed in OA chondrocytes which exhibit a marked decrease in the complexes I, II, and III of the respiratory chain, triggering a reduction in the mitochondrial membrane potential (Δψm) and ATP synthesis (Maneiro et al., 2005). Interestingly, the pro-inflammatory cytokines IL-1β and TNFα, that are found at elevated concentration in the synovial fluid of OA joint, have been shown to alter mitochondrial activity through the reduction of respiratory chain complex I and the decrease in the Δψm and ATP synthesis (Lopez-Armada et al., 2006). In line with these, elevated intracellular levels of ROS and particularly superoxide anion have been found in post-traumatic OA. This superoxide anion accumulation is related to the downregulation of its degrading enzyme, the mitochondrial superoxide dismutase 2 (SOD2) (Koike et al., 2015). Consistently, the mitochondrial dysfunction associated with an imbalance between the ROS production and the antioxidant capacities of the cells is now considered a potent player in the onset and development of OA.

#### Senescent markers in OA

Many of the above reported senescence-associated markers are overexpressed in OA-affected chondrocytes.

##### SAβ-gal

A positive SAβ-gal staining was observed in a subset of chondrocytes close to the lesion sites of mild, moderate and severe OA. Moreover, the level of SAβ-gal correlated with the severity of OA (Gao et al., 2016). Since SAβ-gal staining has been used in many studies in OA (Kim et al., 2014; Nagai et al., 2015; Platas et al., 2016; Jeon et al., 2017), it is now belonging to the conventional arsenal of senescence markers.

##### Telomere shortening

It has been observed that with age, the average telomere length decrease in chondrocytes inducing replicative senescence and participating in the progression of OA (Martin and Buckwalter, 2001). However, since chondrocytes exhibit a poor division rate, telomere shortening may be more likely caused by chronic stress such as oxidative stress known to trigger DNA damage and cell senescence through ROS production (Martin et al., 2004; Davies et al., 2008; Brandl et al., 2011). Nonetheless, during OA the slight reduction of telomere length might be due to the slight increase in chondrocyte proliferation. Recently, a divergent subpopulation of chondroprogenitors with increased telomere erosion has been evidenced in OA cartilage (Fellows et al., 2017).

##### Sirtuins (SIRT)

SIRT1-7 are NAD^+^-dependent deacetylase/deacylases that regulate a wide variety of biological functions. SIRT proteins, through the regulation of energy metabolism, contribute to cellular homeostasis and lifespan (Houtkooper et al., 2012). Among the seven members of the SIRT family, four (SIRT1, SIRT3, SIRT6, and SIRT7) have been studied in articular cartilage or chondrocytes as well as in OA. In the articular cartilage SIRT1 expression decrease with aging and cartilage-specific SIRT1-conditional knockout mice exhibit a more severe OA score at 1 year and an accelerated progression after post-traumatic OA (Matsuzaki et al., 2014b). Moreover, cartilage-specific SIRT1-conditional knockout mice exhibit increased chondrocyte apoptosis and MMP13 and ADAMTS5 expression levels. These data suggest that SIRT1 may have a preventive role in the development of OA via the suppression of the NF-κB pathway activation, a pathway also involved in the induction of SASP (Matsuzaki et al., 2014b). The protective role of SIRT1 was further confirmed by another study demonstrating that SIRT1 gene knock-out may exacerbate cartilage degeneration in OA by activating the SREBP2 protein-mediated PI3K/AKT signaling pathway (Yu et al., 2016). Consistent with this protective role of SIRT1, it has also been shown beneficial effects involving the inhibition of senescence through the activation of autophagy (Lim et al., 2017). Other members of the SIRT family, notably SIRT3, have also been associated with the development of OA. It has thus been demonstrated that SIRT3 is decreased in OA cartilage and associated with a concomitant reduction of SOD2 specificity and activity (Fu et al., 2016a). Consistent with this observation, the genetic ablation of SIRT3 has been found to accelerate the development of OA thereby strongly suggesting a protective role for SIRT3 (Fu et al., 2016a). SIRT6, which is localized to the nucleus and is involved in transcriptional silencing, genome stability, and longevity (Kanfi et al., 2012) has also been suggested as a protective factor in OA. SIRT6 is particularly implicated in the regulation of life span and aging through the regulation of NF-κB signaling and glucose homeostasis. SIRT6 level is significantly decreased in the articular chondrocytes of OA patients (Wu et al., 2015) and is preferentially expressed in cluster-forming chondrocytes of the superficial zone in OA cartilage (Nagai et al., 2015). SIRT6 inhibition is associated with an increased MMP1 and MMP13, decreased proliferation and increased number of SAβ-gal positive chondrocytes. Moreover, the senescent phenotype induced by SIRT6 inhibition has been further confirmed by the increase in P16^INK4A^ expression and the presence of γH2AX foci (Nagai et al., 2015). In a second set of experiments, the overexpression of SIRT6 suppresses the replicative senescence of chondrocytes and reduces the expression of NF-κB dependent genes (Wu et al., 2015). Finally, the lentivirus-associated intra articular delivery of SIRT6 has been shown to protect mice from cartilage degeneration. As a whole, these data strongly suggest that the overexpression of SIRT6 can prevent OA development by reducing both the inflammatory response and chondrocytes senescence. The last SIRT member associated with OA is SIRT7. However, since SIRT7 KO mice are resistant to the development of age-associated OA and forced exercise-induced OA, SIRT7 is believed to exert detrimental effect on cartilage. Whereas its knockdown increases the deposition of a glycosaminoglycan-rich extracellular matrix, SIRT7 has also been shown to suppress the transcriptional activity of SOX9 thereby confirming its putative deleterious effect in OA and cartilage homeostasis (Korogi et al., 2018).

##### HMGB-1

It is localized in the nucleus of non-senescent cells and secreted by senescent cells, making the loss of its nuclear staining a sign of senescence (Davalos et al., 2013). Immunohistochemistry analysis of synovial membrane reveals that HMGB1 expression shift from a strictly nuclear localization in healthy individuals to a nuclear and cytoplasmic localization in OA patients (Ke et al., 2015). Moreover, both the synovium and synovial fluid HMGB-1 levels were significantly higher in OA patients (Ke et al., 2015). In another study, a correlation between the total number of HMGB-1 positive chondrocytes and the OARSI scoring was found (Terada et al., 2011). It has notably been highlighted that in the highest OARSI grade, the number of chondrocytes with cytoplasmic HMGB-1 expression was increased in the deep layers of cartilage. Similarly, the level of expression of the receptor for advanced glycation end product (RAGE), a receptor able to bind HMGB-1, also correlates with the severity of OA (Terada et al., 2011). Of particular interest in an OA context, HMGB-1 is released by chondrocytes or synoviocytes in response to pro-inflammatory cytokines such as IL-1β and TNF-α (Terada et al., 2011; Amin and Islam, 2014; Philipot et al., 2014). These results suggest that HMGB-1, as a pro-inflammatory cytokine, may play a crucial role in the progression of OA. In line with this observation, HMGB-1 is considered an alarmin that are released during tissue damage (Nefla et al., 2016). When secreted, alarmins enhance inflammatory response and catabolic process and participate in the progression of OA. HMGB-1 contains two DNA binding motifs, A and B box. The B box is considered the cytokine active domain of HMGB-1 which is involved in its pro-inflammatory effect, whereas the A box consists in a competitive binding site with antagonist properties. In a recent study, Fu et al. demonstrated that overexpression of HMGB1 A box inhibits the effect of IL-1β on chondrocytes through the suppression of HMGB1/TLR4/NF-κB pathway (Fu et al., 2016b). Recently, the loss of nuclear HMGB1 staining was clearly correlated with an increase in the P16^INK4^ senescent marker expression in post-traumatic OA chondrocytes (Jeon et al., 2017). Of particular clinical interest, the clearance of senescent cell was found to reestablish the nuclear staining of HMGB1 (Jeon et al., 2017) in the same post-traumatic OA models.

##### CAV1

CAV1 as described in the section Identification of Senescent Cells is involved in the senescence of various cell type (Zou et al., 2011). In chondrocytes, IL-1β and H_2_O_2_, both senescence inducer, increase CAV1 expression, and promote senescence phenotype such as telomere erosion, SAβ-gal activity and altered morphology through activation of the P38MAPK pathway (Dai et al., 2006; Yudoh et al., 2009). All these senescence-associated phenotypic alterations induced by IL-1β and H_2_O_2_ are consistently inhibited by an antisense oligonucleotide targeting CAV1 (Dai et al., 2006). These results strongly suggest that CAV1 is involved in IL-1β-induced senescence in chondrocytes. Finally, increased expression of CAV1 has also been observed in OA cartilage samples and is correlated with the disease severity (Min et al., 2015). However, considering the contradictory data regarding the effect of CAV1 overexpression or deficiency on senescence in other organs, the role of CAV1 in OA cartilage needs further investigations notably through the use of cartilage specific CAV1 conditional knockout mice.

##### P16^*INK*4*A*^

It is physiologically involved in the processes of cartilage aging, and may be partly responsible for the senescence of chondrocytes as seen in OA (Zhou et al., 2004). Indeed, a significant increase in p16^INK4a^ was detected in OA chondrocytes as compared to age-matched healthy chondrocytes *in vivo* and *in vitro* (Zhou et al., 2004). Moreover, P16^INK4^ silencing in OA chondrocytes has been found to increase chondrocyte proliferation and DNA synthesis and to decrease SAβ-gal staining (Zhou et al., 2004). The expression of P16^INK4A^ has been also associated with the terminal differentiation of chondrocytes *in vitro* (Philipot et al., 2014) and IL-1β treatment of human OA chondrocytes induce the expression of P16^INK4A^, which leads to an increased production of OA-associated catabolic proteases MMP1 and MMP13 (Philipot et al., 2014). Recently, the causal role of P16^INK4A^ positive cells in the OA onset has been demonstrated. Through the use of the P16-3MR transgenic mice, the kinetics of senescence after OA surgical induction was investigated. Using this murine model as well as the senolytic compound UBX0101, they undoubtedly demonstrate the beneficial effect of removing p16^INK4A^ positive senescent cells in OA, as evidenced by a reduction in cartilage damage and the promotion of cartilage repair (Jeon et al., 2017).

##### SASP

Senescent cells produce a bioactive secretome which participate in the propagation of senescence to neighboring cells (Hoare and Narita, 2013). Numerous SASP components described in senescent cells are also present in OA tissues or synovial fluid (Kapoor et al., 2011). The implication of some SASP factors in OA will be addressed below. The proinflammatory cytokines IL-6, IL-8, TNF-α, IL-1α, and IL-1β are particularly relevant in OA (Robinson et al., 2016). These cytokines have been shown to promote OA progression, notably by altering chondrocytes function and viability and by inducing synovitis (Kapoor et al., 2011). IL-1β and TNFα drives chondrocytes toward a senescent phenotype (Philipot et al., 2014) comprising the secretion of a SASP containing IL-6, IL-8, MMP-13, MMP-1, MMP-9, MMP-3, and MMP-14, ADAMTS4, 5, and 9 as well as PGE2 and NO which participate in pathogenesis. IL-1β and TNFα are the most widely used cytokines to induce an OA-like phenotype in chondrocytes *in vitro* (Fukui et al., 2003; Carames et al., 2008) and are now recognized as senescent inducers (Platas et al., 2016; Song et al., 2018). The effect of IL-1β and TNFα are mediated by the activation of p38 MAPK and NF-κB signaling pathways (Ovadya and Krizhanovsky, 2014). IL-6 level is increased in synovial fluid of OA patients (Kaneko et al., 2000) and IL-6 production is directly stimulated by IL-1β and TNF-α in chondrocytes (Guerne et al., 1990). However, the role of IL-6 in OA is controversial. On the one hand IL-6 upregulates MMP-1 and MMP-13 expression, and on the other hand, IL-6 intra articular injection in IL-6 deficient mice reduced cartilage degeneration (van de Loo et al., 1997). This opposite effect of IL-6 cytokine may be due to the type of IL-6 receptor engaged to mediate its signaling (membrane-bound or soluble form; Hunter and Jones, 2015). IL-8 is significantly increased in the synovial fluid of OA patients and induces chondrocytes hypertrophy (Takahashi et al., 2015). Matrix degrading enzymes such as a disintegrin and metalloproteinase with thrombospondin motifs (ADAMTS4 and ADAMTS5) and Metalloproteinases (MMP-1, MMP2, MMP-3, MMP-14) are also involved in OA pathogenesis. MMP-13 and MMP-1 are secreted by IL-1β-induced senescent chondrocytes, which promote the degradation of aggrecan and type II collagen (Philipot et al., 2014). ADAMTS4 is also induced by IL-1β and TNFα in chondrocytes. Many chemokines are produced in OA, but CCL2/MCP-1 is considered a major component of the SASP. CCL2 indeed up-regulates the expression of MMP13 and increase proteoglycan loss *in vitro*. MCP-1 synovial fluid level is positively correlated with OA severity and pain (Miller et al., 2012; Li and Jiang, 2015). In line with this observation, mice lacking CCL2 or its receptor CCR2 have recently been found protected against OA (Raghu et al., 2017). VEGF is also a common component of the SASP, and VEGF, which is expressed in OA cartilage, participate in the dysregulation of bone remodeling through promoting osteophytes formation.

Reinforcing the idea of the production of a SASP by chondrocytes during OA, the NF-κB pathway described above as the main pathway governing the SASP production (Chien et al., 2011) is also an important signaling pathway in OA. Indeed, several studies reporting beneficial anti-inflammatory effects of drugs on OA are mediated through the inhibition of the activation of NF-κB pathway.

Specially promising are the beneficial effects obtained by selective removal of the senescent chondrocytes from OA patients, which decreases expression of senescent and inflammatory biomarkers and increases expression of ECM proteins (Jeon et al., 2017). There are in fact progenitors with senescence phenotypic characteristics (β-galactosidase activity and telomere shortening) derived from osteoarthritic cartilage (Fellows et al., 2017). Together, these preclinical data support the concept of selective targeting senescent chondrocytes as a novel therapeutic strategy for OA.

## Autophagy in OA

### General context

Autophagy is the cellular mechanism that degrades proteins and organelles to maintain cellular homeostasis and quality control (Sridhar et al., 2012). Physiological functions such as remodeling, differentiation, survival, death, senescence, and longevity are regulated by autophagy (Cuervo and Macian, 2012). The immune response is also regulated by autophagy in infection, inflammation and adaptive immunity, degrading intracellular pathogens, and presenting antigens and activating lymphocyte proliferation (Deretic et al., 2013; Arroyo et al., 2014). In normal conditions, autophagy constitutively occurs in all mammalian cells, and can be regulated as a consequence of stresses such as nutrient deprivation, heat, oxidation, and infection (Meijer and Codogno, 2009). Autophagy involves multiple steps including initiation, nucleation, elongation, maturation, and degradation. The steps involving the autophagosome formation result in the enzymatic degradation of the sequestered products that are transformed into nucleotides, amino acids, fatty acids, and sugar, which can be recycled into metabolic pathways to generate energy and build macromolecules (Glick et al., 2010). These rate-limiting steps are mediated by the autophagy-related genes (ATGs) (Mizushima et al., 2011; Abounit et al., 2012). Uncoordinated-51-like kinase (ULK) complex, composed by ULK1/2, Atg13, Focal Adhesion Kinase (FAK), Family Interacting Protein of 200 kDa (FIP200) and ATG101, regulates the induction of the autophagosome formation. This complex is the final target of Mammalian Target of Rapamycin (mTORC1) and Adenosine 5′Monophosphate-Activated Protein Kinase (AMPK) signaling cascades (Wesselborg and Stork, 2015). mTORC1 is the main inhibitory signal of autophagy, and is regulated by growth factors, energy levels and nutrient availability, among other stress signals. Signaling converges through Phosphatidylinositol 3-Kinase (PI3K), Mitogen-Activated Protein Kinase (MAPK), and p90 Ribosomal S6 kinase (RSK) pathways, each one inhibiting Tuberous Sclerosis Heterodimeric Complex (TSC1/TSC2) complex. TSC1/TSC2 complex inhibition will subsequently stimulate mTORC1 (Sengupta et al., 2010). In respect to energy status, the decreased ATP levels activate AMPK, which in turn activates the TSC1/TSC2 complex, resulting in mTORC1 inactivation (Wullschleger et al., 2006). Indeed, nutrient rich-conditions activate mTORC1 that conjugates with ULK complex, avoiding the conjugation of ULK with AMPK, and results in autophagy inhibition. On the other hand, in energy depletion and starvation, mTORC1 dissociates from ULK, allowing the association between AMPK and ULK which consequently results in autophagy induction (Papinski and Kraft, 2016). The nucleation is mediated by PI3K, composed by a central core complex resulting from the association between class III PI3K (Vacuolar Protein Sorting Protein 34, hVps34), beclin-1 and p150. This complex is responsible to the expanding phagophore (Funderburk et al., 2010). The elongation and enclosure of the autophagosome involves 2 ubiquitin-like conjugation systems: Atg12 and LC3/Atg8. These two conjugation systems are essential for autophagosome formation (Geng and Klionsky, 2008; Glick et al., 2010). Accumulation of undegraded substrates on lysosomes may have a great impact on cells and tissues, highlighting the importance of lysosome dysfunction and impaired proteostasis as a major outcome of failure of autophagy and a key feature of senescence. This particularly relevant in metabolic tissues such as liver and heart (Schneider et al., 2015; Gianfranceschi et al., 2016), but might also be true in musculoskeletal tissues.

### Relevance of autophagy in articular cartilage and OA

Autophagy is essential to preserve the integrity and function of articular cartilage. Normal human cartilage expresses high levels of autophagy regulators, including ULK1, Beclin1, and LC3-II, suggesting that autophagy is a constitutively active mechanism in cartilage. Seminal studies in human OA cartilage and in an experimental OA, demonstrated that autophagy is abnormally reduced (Carames et al., 2010). Consistent with these findings, mTOR was overexpressed in human OA cartilage and mouse models of experimental OA (Zhang Y. et al., 2015). Moreover, a positive correlation of mTOR activation in both peripheral blood mononuclear cells (PBMC) and articular cartilage was found in end-stage OA patients (Tchetina et al., 2013; Zhang et al., 2017). On the other hand, LC3-II and Beclin1 are upregulated in OA chondrocytes (Sasaki et al., 2012), while OA tissues display numerous LC3 puncta via hypoxia-inducible factor 2 (Bohensky et al., 2009). A potential explanation could be the different location of harvested OA cartilage potentially corresponding to different disease stages (cartilage from lateral femoral condyles was identified as mild OA, while cartilage from medial femoral condyles was identified as severe OA), where autophagy markers were differentially expressed within these two regions. Mild OA cartilage had a strong expression of autophagy markers compared to non-OA cartilage and severe OA cartilage. During OA progression, autophagy may act as an adaptive response in an attempt to protect cells. However, once severe OA is established, a decrease in autophagy is detected and could eventually contribute to OA progression (Sasaki et al., 2012).

A similar dual role of autophagy can be seen regarding its effect on cell death. Many authors consider the activation of autophagy as a protective mechanism that avoids chondrocyte death (Carames et al., 2012a,b; Sasaki et al., 2012), while others propose that reduction of autophagy is often accompanied with increased apoptosis (Carames et al., 2010; Zhang Y. et al., 2015). Moreover, the up-regulation of autophagy suppresses glucocorticoid-stimulated chondrocyte apoptosis (Liu et al., 2014), paradoxically constituting an alternative form of cell death generally observed in different pathological disorders (Levine and Yuan, 2005; Maiuri et al., 2007). Indeed, exposure to monosodium urate crystals promotes chondrocyte death through autophagy activation (Hwang et al., 2015). Young chondrocytes are protected from cell death in OA chondrocytes (Chang et al., 2013). Autophagy might play a possible dual role at different stages of progression: protective and a death-promoting in OA pathogenesis, where the death of chondrocytes appears to be associated to a complex interaction between autophagy and apoptosis across different stages of OA as well as different anatomical locations (Almonte-Becerril et al., 2010). These conflicting results may suggest that autophagy could promote either chondrocyte survival or death depending on donor age, the presence and stage of OA, and the type of autophagy induction (Hwang et al., 2015). In fact, this dual role of autophagy is consistent with studies in other cell types and tissues, mainly dependent on the type of stress stimuli (Chen et al., 2010). Notwithstanding, the relationship between autophagy and cell death is not fully understood and thus prompts additional studies to decipher the underlying cell signaling mechanisms (Musumeci et al., 2015).

There is a consensus at recognizing the protective role of autophagy under stress conditions. Both nutritional (starvation) and catabolic stresses (IL-1β or sodium nitroprusside, SNP) increase autophagy in chondrocytes (Sasaki et al., 2012). In cartilage challenged with mechanical injury, initial upregulation of LC3-II is detected at 24 h, while 48 and 96 h after injury, LC3-II levels appear to decrease (Carames et al., 2012b). Similarly, the biomechanical dental stimulation that leads to degradation of the cartilage from the temporomandibular joint also increases autophagy as an early response (Zhang et al., 2013). Intermittent cyclic mechanical tension (ICMT) leads to calcification of end plates of the intervertebral disc, which is responsible for its degeneration. Short-term ICMT increased autophagy and it was accompanied by an insignificant calcification of end plate chondrocytes. However, long term ICMT suppressed autophagy, leading to endplate chondrocyte calcification. Although the cartilage of intervertebral disc differs from articular cartilage, a similar mechanism may be involved (Xu et al., 2014; Xu, 2015). Furthermore, in response to energy stress through mitochondrial dysfunction, chondrocytes showed an early increase in autophagy as a compensatory mechanism. However, when prolonged stress exceeds cellular compensation, damage occurs (Lopez de Figueroa et al., 2015). Both energy and mechanical stress activate autophagy initially, but this is probably insufficient to protect cartilage in the long-term and eventually autophagy becomes defective. These results provide strong evidence that autophagy has an important role in protecting chondrocytes from different stressors and, therefore, can be involved in OA pathogenesis.

### Major targeted pathways to elucidate the relevance of autophagy in articular cartilage and OA

#### PI3K/AKT/mTOR

mTOR is been extensively studied as a key autophagy regulator in OA. Inducible cartilage-specific mTOR KO mice subjected to experimental OA by destabilizing the medial meniscus (DMM model) have less cartilage degradation, more proteoglycans and chondrocytes, as well as less signs of synovial fibrosis. Autophagy markers (i.e., ULK1, AMPK1, Atg5, LC3) are significantly increased, while apoptotic cells and catabolic factors, such as MMP-13 and MMP-induced type II collagen breakdown product C1, 2C, are reduced (Chen et al., 2013). In human disc cells, mTORC1/RAPTOR silencing protects against inflammation through AKT and autophagy induction (Ito et al., 2017). An interesting link between mTOR pathway and hormonal regulation was found in cartilage-specific Tsc1 KO and inducible Tsc1 KO mice, where articular chondrocyte proliferation and differentiation are activated to initiate OA, in part by downregulating FGFR3 and PPR (Zhang et al., 2017). Sestrins, a family of highly conserved stress-responsive proteins that are transcriptionally regulated by p53 and forkhead transcription factor (FoxO), protect cells from stress conditions by regulating AMPK and mTOR signaling. In aging and OA cartilage, sestrins are suppressed and promote autophagy by inhibiting mTOR, contributing to reduce homeostasis (Shen et al., 2017). Regulated in development and DNA damage response 1 (Redd1), an inhibitor of mTOR signaling, is regulated by ubiquitin ligases and is highly expressed in normal human cartilage and reduced in aging and OA (Alvarez-Garcia et al., 2016). In mice lacking Redd1, autophagy and mitochondrial biogenesis are reduced, which leads to OA (Alvarez-Garcia et al., 2017). The regulatory role of mTOR in autophagy also control inflammatory responses and can prevent both cartilage damage and OA (Salminen et al., 2012; Rahmati et al., 2016). Increased mTOR expression in peripheral blood mononuclear cells (PBMC) was associated with synovitis (Tchetina et al., 2013). In inflammatory arthritis *in vivo*, mTOR inhibition reduced osteoclast numbers and activity, protected against local bone erosions and cartilage damage, and decreased synovitis (Cejka et al., 2010). A similar association was observed in OA, where mTOR deletion reduced synovial inflammation and decreased IL-1β expression (Carames et al., 2012a). Moreover, chondrocytes from PPARγ KO mice showed an increase in inflammatory mediators (i.e., COX-2 and iNOS), enhanced expression of mTOR and a decrease in autophagy markers (Vasheghani et al., 2015). On the other hand, human OA chondrocytes treated with IL-1β show increased expression of mTOR in conjunction with increased expression of catabolic factors and a decreased expression of collagen type II, suggesting that pro-inflammatory cytokines can also alter mTOR pathway in OA (Zhang Y. et al., 2015). Viewed together, these studies suggest an important connection between mTOR, inflammation and cartilage damage.

#### Nuclear receptors and transcription factors

PPARγ plays a protective role in articular cartilage. PPARγ KO mice exhibited increased apoptosis as well as production of inflammatory and catabolic factors, and decreased expression of anabolic factors, resulting in an accelerated OA (Vasheghani et al., 2013, 2015). A significant reduction in LC3-II and increased mTOR expression were detected in those animals, while in chondrocytes, the restoration of PPARγ expression downregulates mTOR and up-regulates LC3-II expression. Catabolic and inflammatory factors are decreased, while anabolic factors are increased. PPARγ-mTOR double KO mice are protected from experimentally induced-OA. Degradation of cartilage is less severe, less proteoglycan and chondrocyte loss is observed, and these beneficial effects are associated with increased LC3-II and reduced MMP-13 expression. Thus, decreased PPARγ contributes to mTOR compensatory up-regulation that is responsible for autophagy suppression, lead to chondrocyte death and increased catabolic activity, which ultimately accelerates OA. Both *in vivo* studies confirm that mTOR deletion protects against OA, thus reinforcing the role of decreased autophagy in OA development.

Impressive proof-of-concept in the context of cartilage biology and OA has recently been shown for the role of FoxO transcription factors as major regulators of autophagy, metabolism and aging of the joint (Matsuzaki et al., 2018). FoxO plays a key role in development, aging and longevity. Articular cartilage degradation is caused by age, genetic and environmental challenges, which ultimately lead to OA. In the absence of insulin and growth factor signaling, FoxO are translocated to the nucleus and result in the integration and activation of a cascade of key target genes that cause cell cycle arrest, stress resistance, and cell death (Webb and Brunet, 2014). FoxOs trigger autophagy in a variety of tissues, including skeletal muscle and cartilage. Human chondrocytes from patients suffering OA have FoxO1 and ATGs reduced, and restoring FoxO1 decreased inflammatory cytokines and up-regulated lubricin, a secreted proteoglycan that contributes to the lubrication and minimize friction of joint synovium. In this study, Matsuzaki and collaborators demonstrated the importance of FoxO on postnatal cartilage development, maturation, and homeostasis of cartilage. In young mice lacking FoxO1/3/4, cartilage was thicker and chondrocytes were more proliferative. Chondrocyte-specific FoxO-deficient mice exhibited severe joint damage with aging and increased cartilage degradation in response to surgically induced OA. More importantly, expression of superficial zone protein PRG4, was significantly reduced. Further studies to investigate the molecular mechanism of FoxO on cartilage superficial zone homeostasis indicate that FoxO1 is a transcriptional factor of PRG4 in cartilage. *In vitro* studies in IMACS and ATDC5 chondrogenic cells showed that overexpression of FoxO1 significantly up-regulates the expression of PRG4. Moreover, previous studies shown reduced FoxO in aging and OA (FoxO 1, 3, and 4) associated with increased susceptibility to cell death induced by oxidative stress and reduced levels of antioxidant proteins as well as autophagy (Akasaki et al., 2014a,b). Thus, these results indicate the importance of FoxO in aging and OA and suggest that targeting FoxO transcription could be a novel strategy to prevent or delay OA progression.

p63 belongs to p53 family and play an important role in tissue development. Global and tissue-specific overexpression of p63α and p63γ mice with aging or surgically induced instability showed significant resistance to OA development and suppression of chondrocyte apoptosis (Taniguchi et al., 2017). Proapoptotic genes were increased in chondrocytes along with in the growth plate. In contrast, p53 was more predominant in the superficial zone of articular cartilage, as opposed to p63. Immunosuppressive drugs have deleterious effects on growth and senescence of articular chondrocytes from rabbit (Kang et al., 2016).

#### Aging-related autophagy

Aging is one of the major risk factors for OA, precipitating onset predominantly among adults 60 years of age or older (Lotz and Loeser, 2012). The low turnover of ECM and articular cartilage likely increases the sensitivity to accumulate age-related changes, including a disturbed structural organization of ECM due to formation and accumulation of AGEs, cartilage calcification, and fibrosis, which alter cartilage mechanical properties (Roberts et al., 2016). Cellular age-related changes, those include senescence, reduction in the number of chondrocytes, mitochondrial dysfunction, and altered growth factors responsiveness. Alterations in protective mechanisms such as decreased antioxidant defense and reduced autophagy also occur with aging, disturbing the anabolic-catabolic equilibrium and reducing the remodeling and repairing ability of cartilage (Lotz and Loeser, 2012; Hui et al., 2016). Autophagy can regulate age-related changes in articular cartilage. Both aging human and mice chondrocytes exhibit a reduction in constitutive autophagy. Moreover, compromised autophagy as a consequence of aging precedes the decrease in cartilage cellularity and the onset of structural damage (Carames et al., 2010, 2015; Hui et al., 2016). Chondrocyte-specific ablation of autophagy gene Atg5 in mice leads to age-related OA but no alteration of injury-induced OA (Bouderlique et al., 2016). Altered tissue repair, misbalanced homeostasis and defective autophagy are important hallmarks of aged tissues in general (Cuervo et al., 2005; Cuervo, 2008). Consistently, the molecular mechanisms occurring during aging can explain the impairment in autophagy, including defects in induction and inefficient lysosomal clearance. For instance, the altered hormonal regulation of autophagy occurring in aged tissues can be a possible explanation for the compromised autophagy observed in old organisms caused by glucagon upregulation (Cuervo et al., 2005). Signaling pathways involving pro-longevity factors such as Sirtuin 1 (SIRT1), transcription factor forkhead-box O3 (FOXO3), and pro-senescence factors NF-kB and p53 are also known autophagy regulators and thus can be involved in cartilage aging (Salminen and Kaarniranta, 2009). Decreased autophagy can be explained by the impaired capacity of the lysosomes to fuse with autophagosomes and the failure of lysosome hydrolases that decrease the proteolysis efficiency of lysosomes (Cuervo, 2008). In fact, the accumulation in the lysosomes of undegradable materials that occurs with aging hampers the degradative capacity and results in decreased autophagy (Brunk and Terman, 2002a,b; Terman and Brunk, 2004a).

In cardiac and skeletal muscle of mice and humans with progeria, autophagy decreases with aging, while mTOR activity is elevated (Ramos et al., 2012; Sandri et al., 2013). Pharmacological and genetic inhibition of mTOR extends lifespan by unknown mechanisms (Lamming, 2016). mTOR inhibition represses autophagy promoting the degradation of aberrant proteins and damaged organelles, thus protecting from toxicity, and consequently slowing aging. Other processes including the regulation of protein synthesis, regulation of mitochondrial function, anti-inflammatory effects, increased stress resistance and preservation of stem-cells might also contribute to the pro-longevity effects of mTOR inhibition (Johnson et al., 2013). Aging and OA are clearly intertwined, and autophagy might be a common mechanism involved in cartilage degradation in both conditions.

Kashin-Beck is a rare disease of the bone occurring in children, with high prevalence in certain areas of Asia. Functional genetic studies identified ATG4C as a novel susceptibility gene in Kashin-Beck (Wu et al., 2014). In normal human chondrocytes stimulated with IL-1β, Parkin, a component of the multiprotein proteasome complex, eliminates dysfunctional mitochondria promoting survival and protecting from apoptosis (Ansari et al., 2017). In age-related and surgical OA mice, Bach1 deficiency reduces the severity of articular cartilage damage probably by antioxidant effects of HO-1 and downregulation of ECM catabolic enzymes (Takada et al., 2015). Chondrocyte-derived extracellular organelles from adult porcine and osteoarthritic patients evaluated by light scatter nanoparticle counting have constitutive autophagy that is increased with rapamycin and suppressed by autophagy inhibitors and genetic silencing of ATG5, supporting the role of autophagy in cartilage disease and repair (Rosenthal et al., 2015).

#### microRNA

Small non-coding RNA molecules have been extensively used as tools to study the post-transcriptional regulation of gene expression. miRNA have been manipulated in mice, articular cartilage and chondrocytes to test different hypothesis related to musculoskeletal system physiology and disease. In OA model mice and SW1353 human chondrosarcoma cells treated with interleukin-1β, miR-17-5p was decreased (Li et al., 2018). Autophagy was found suppressed in knee joints mainly through suppressing p62/SQSTM1, an autophagosome cargo protein that targets other proteins that bind to it for selective autophagy. In an hypoxic environment as occurs in aged articular cartilage, miR-146a targets related to inflammation Traf6 and IRAK1, as well as chondrocyte-specific Smad4 were down-regulated (Chen et al., 2017). Traf6 and IRAK1 were identified as regulators of autophagy. Previous reports found that miR-146a promote chondrocytes autophagy via depressing Bcl-2 (Zhang F. et al., 2015). In primary human and TC28a2 chondrocytes, miR-155 inhibited autophagy and contributed to the autophagy defects in OA (D'Adamo et al., 2016). In rats with OA, miR-4262 regulates chondrocyte fate by influencing PI3K/AKT/mTOR signaling (Sun et al., 2018). In OA chondrocytes and in zebra fish, Fis1 suppression induces accumulation and inhibition of lysosomes by altering miRNAs and energy signals (Kim et al., 2016). These results indicate that post-transcriptional targeting of autophagy with specific miRNAs might be considered as a promising preclinical model to study musculoskeletal physiology and disease.

These pathways span a broad range of biological processes, and therefore targeting specific pathways with small molecules should be taken into consideration regarding the wide endogenous functions of these pathways in non-target tissues. Table 1 summarizes relevant advances in the autophagy-related molecular mechanisms in OA.

**Table 1:** Recent relevant advances in the autophagy-related molecular mechanisms in OA.

**Targeted pathway**	**Molecular mechanism**	**Relevant phenotype**	**Model**	**References**
PI3K/AKT/mTOR	mTORC1/RAPTOR	Selective interference of mTORC1/RAPTOR protects against inflammation senescence and matrix catabolism through AKT and autophagy activation	Human disc cells	Ito et al., 2017
	mTORC1	mTORC1 induces OA by reducing FGFr3 and PTH/PTHrP	OA human cartilage and surgically-induced OA mice	Zhang et al., 2017
	mTOR	Cartilage-specific mTOR KO up-regulates autophagy and protects against OA	OA human cartilage and mouse/dog experimental OA	Zhang Y. et al., 2015
	mTORC1 inhibitor	Diabetes-accelerated experimental OA is prevented by autophagy activation	Experimental OA mouse model	Ribeiro et al., 2016b
	Sestrins	Sestrins is reduced in OA cartilage, promotes chondrocyte survival under stress and promotes autophagy by modulating mTOR	Human and mouse normal and OA cartilage	Shen et al., 2017
	Redd1	Redd1 is highly expressed in normal human cartilage and reduced in Aging and OA	Human and mouse normal, aging, and OA cartilage	Alvarez-Garcia et al., 2016, 2017
		Redd1 deficiency reduce autophagy and mitochondrial biogenesis in articular cartilage and increase OA severity in mice	Experimental OA mouse model	
Nuclear receptors/transcription factors	PPAR-gamma	PPAR-gamma maintain cartilage homeostasis by regulating mTOR pathway	Experimental OA Mouse Model	Vasheghani et al., 2015
	FoxO	Reduced FoxO increased cell death by oxidative stress and reduced autophagy	Human chondrocytes	Akasaki et al., 2014a
	FoxO 1,3,4	Reduced FoxO in Aging and OA	Human and mouse normal, aging and OA cartilage	Akasaki et al., 2014b
	FoxO 1,3,4	FoxO transcription factors are critical for cartilage maturation, homeostasis and OA	Mutant mice/surgical and treadmill OA models	Matsuzaki et al., 2018
	p16	Local clearance of senescent cells attenuates the development of post-traumatic OA	Mutant Mice/Experimental OA	Jeon et al., 2017
	Telomeres	Progenitor cell sub-populations in OA cartilage:Telomere erosion/replicative senescence	Normal and OA Human Cartilage	Fellows et al., 2017
	p53	Paracrine effects of human MSC in inflammatory-induced senescence features of OA	OA human chondrocytes	Platas et al., 2016
	p63	Regulation of chondrocytes survival in mouse articular cartilage	Mutant mice/aging and surgical OA model	Taniguchi al., 2017
	p38	Inhibition of senescence and proliferation of chondrocytes by inhibition of p38MAPK	Rabbit chondrocytes	Kang et al., 2016
Aging-related autophagy	Kashin-Beck	Defective autophagy in chondrocytes from KB chondrocytes but higher than OA	Human chondrocytes	Wu et al., 2014
	Parkin	Elimination of Parkin damage mitochondria in IL-1b-stimulated chondrocytes	OA human chondrocytes	Ansari et al., 2017
	Batch1	Batch-1 deficiency reduces OA severity by increasing HO-1	Mutant mice/aging and surgical OA model	Takada et al., 2015
	Autophagy	Autophagy modulates articular vesicles formation	Healthy and OA human chondrocytes	Rosenthal et al., 2015
	Oxidative Changes	Oxidative changes are pivotal in iniciating age-related changes in articular cartilage	Aging mouse model	Hui et al., 2016
	Atg5	Genetic deletion of Atg5 in chondrocytes promotes age-related osteoarthritis	Mutant mice and surgical OA	Bouderlique et al., 2016
miRNAs	miRNA-17-5p	Inhibition of miRNa-17-5p increases autophagy (LC3) and reduce p62	OA mouse model/SW1353 cell line	Li et al., 2018
	miRNA-146a	Hypoxia and miRNA-146a induces autophagy via Traf6 and IRAK target genes	Chondrocytes	Chen et al., 2017
	miRNA-146a	Hypoxia and miRNA-146a increase autophagy by depressing Bcl-2	Chondrocytes	Zhang F. et al., 2015
	miRNA-155	Overexpression of miRNA-155 contributes to autophagy defects in OA	Human chondrocytes/T/C28a2 cell line	D'Adamo et al., 2016
	miRNA-4262	Regulates chondrocyte viability, autophagy by targeting SIRT1 and PI3K/AKT/mTOR	Rat chondrocytes	Sun et al., 2018
	Fis1	Fis1 suppression impairs lysosomal function, increases apoptosis, reduces autophagy	Normal and OA human chondrocytes/zebrafish model	Kim et al., 2016

## Current development in innovative therapeutic approaches

Although many advances to understand the pathophysiological processes have been made, no effective treatments to stop or prevent OA exist. Few symptomatic treatments are available, mainly focusing on pain relief and improving joint function. Current clinical management relies in a combination of pharmacological approaches with surgical procedures. Also, decisions on treatment greatly depend on different patient-related factors, such as the occurrence of other co-morbidities and the presence of one or multiple affected joints. Pharmacological options include analgesic and anti-inflammatory agents primarily. When the goals are not achieved with conservative treatment and the disease progresses to diminished quality of life, surgical options are considered. However, the burden, risks and lack of long-term efficacy that come with surgical interventions, ultimately may affect the quality of life of patients and impact the health care system. Thus, efforts focused on generating strong preclinical evidence that can lead to the development of novel targeted therapies are necessary.

### Approaches targeting autophagy as OA therapeutics

Pharmacological activation of autophagy with small molecules has been the subject of several studies showing in some cases some preclinical efficacy in OA (Table 2). Targeting PI3K/AKT/mTOR pathway to modulate autophagy stands out as a major focus of pharmacological interventions in preclinical studies. Rapamycin induces autophagy activation through the inhibition of mTORC1 in many cell and tissues (Galluzzi et al., 2017). It is used as an immunosuppressive in transplantation and autoimmune disorders, and in cancer due to its activity at inhibiting growth and proliferation of tumor cells (Lamming et al., 2013). In articular cartilage, mTOR inhibition by Rapamycin protects from oxidative stress, and cell death (Carames et al., 2012b; Sasaki et al., 2012; Lopez de Figueroa et al., 2015), increases the expression of aggrecan and type II collagen, while decreases MMP-13 in OA chondrocytes (Zhang Y. et al., 2015). C57Bl/6J mice with experimentally induced OA treated with Rapamycin show autophagy activation, including increased LC3-II levels and inhibited rpS6 phosphorylation in articular cartilage. Treatment also protects against OA-like alterations, including the maintenance of cartilage cellularity and prevention of ECM damage. Furthermore, the severity of synovitis was attenuated and inflammatory cytokine IL-1β was decreased. These results suggest that induction of autophagy can significantly reduce the severity of experimental OA (Carames et al., 2012a). It is interesting to note that beneficial effects are also observed in meniscus, a specific type of cartilage not present in all human joints (Meckes et al., 2017). However, systemic long-term administration of Rapamycin is associated with side effects including edema, mucositis, hair and nail disorders, and dermatological effects, among others, making its utility problematic for chronic conditions (Lamming et al., 2013). In order to avoid these side effects, it has been suggested the intra-articular injection as a more appropriate route for clinical use. Indeed, intra-articular injection reduces articular degeneration and preserves hyaline cartilage, by downregulating cartilage degrading enzymes (MMP9 and MMP13), hypertrophy-related genes (vascular endothelial growth factor-VEGF- and COL10A1), inflammation-related genes (IL-1β and IL-6), and stress-responsive genes (CCAAT/enhancer binding protein beta-C EBPβ and mTOR) and by upregulating Col2a1 (Matsuzaki et al., 2014a; Takayama et al., 2014). These studies show that direct administration of autophagy activators in the joint have protective effects, while potentially avoiding the side effects observed with systemic administration. Selective ATP-competitive inhibition of mTOR with Torin1 blocks phosphorylation of both mTORC1 and mTORC2 and induces autophagy by blocking signals required for cell growth and proliferation (Thoreen et al., 2009). Torin 1 treatment reduces severity of OA pathology in rabbits (Cheng et al., 2016a,b), highlighting again the important role of PI3K/AKT/mTOR in articular cartilage degeneration. However, it is important to consider the negative feedback loop of the PI3K/Akt pathway. mTOR signaling regulates protein synthesis and autophagy mediated, at least in part, through changes in the phosphorylation of downstream molecules such as p70S6K, 4E-BP1, and ULK1. Ultimately, the cascade leads to the inhibition of the insulin receptor substrate (IRS1), an upstream regulator of PI3K/Akt (Laplante and Sabatini, 2012). On the other hand, mTOR inhibition leads to an enhancement of PI3K/Akt signaling and increased MMP production by chondrocytes. Based on this, Chen et al. proposed the dual inhibition of both pathways as a more promising approach for OA to abolish the negative feedback mechanism and its possible side effects (Chen et al., 2013). On the other hand, excessive inhibition of mTOR gene expression could be harmful for tissues. OA outpatients with lower mTOR gene expression in PBMCs compared to healthy controls, exhibited more pain while standing and upon joint function, and have increased total joint stiffness than OA outpatients with higher mTOR gene expression (Tchetina et al., 2013). Inhibition of PI3K/AKT/mTOR promotes autophagy and attenuates inflammation in rat articular chondrocytes (Xue et al., 2017). Chronic upregulated autophagy has been associated with compromised lifespan and pathology. For instance, mouse models of premature aging show systemic degeneration and weakening of the musculoskeletal system partly due to DNA damage defects (Marino et al., 2008). Therefore, continuous autophagy activation might not likely be the best therapeutic approach. Instead, repairing autophagy defects, or restoring the normal levels of autophagy, could be a more efficient therapeutic strategy for age-related diseases. Sugars, as precursors of the synthesis of glycosylated proteins and lipids, have also been tested as therapeutic agents for OA. Glucosamine is a dietary supplement marketed to support the structure and function of joints. In chondrocytes and in articular cartilage, it protects nucleus pulposus cells through the inhibition of mTOR and consequent activation of autophagy (Carames et al., 2013; Jiang et al., 2014). Primary human chondrocytes treated with sucrose are protected from degradation, chondrocyte inflammation and death via PI3K/Akt/mTOR (Khan et al., 2017), and trehalose reduces ER and oxidative stress-mediated by autophagy stimulation (Tang et al., 2017). Although these results are promising, the time-dependent dual role of glucosamine (short-term exposure increases autophagy, while long-term exposure have inhibitory effects (Kang Y. H. et al., 2015), making these data difficult to interpret. Interestingly, drugs widely used in the management of OA such as glucocorticoid dexamethasone have also shown a similar dual profile of action in chondrocytes via FOXO3 and in meniscus via inositol 1,4,5-trisphosphate receptor (Liu et al., 2014; Shen et al., 2015a,b). Perhaps, autophagy activation is an early protective response, whereas prolonged treatments eventually lead to permanent autophagy defect. These dual effects might be due to the particular metabolism and pharmacokinetics of these molecules and the wide interference with diverse metabolic and inflammatory pathways. Whether this dual effect is generally observed with such molecules potentially interfering with autophagy, remains to be clarified. Glucose metabolism might play a relevant role in cartilage as diabetic mice with experimental OA have is degenerated joint tissue and Rapamycin treatment decreases both pathology and inflammation (Ribeiro et al., 2016b). Indeed, insulin might exert catabolic effects in chondrocytes, inducing loss of proteoglycans and inflammation by downregulating autophagy, similarly to what is observed in chondrocytes from diabetic-OA patients (Ribeiro et al., 2016a). On the other hand, sirtuins have arisen as potential targets of phenol-like molecules such as Hydroxytyrosol to prevent inflammation by promoting cell autophagy (Cetrullo et al., 2016; Zhi et al., 2018).

**Table 2:** Preclinical treatments targeting autophagy in OA.

**Type of preclinical OA treatment**	**Name**	**OA Efficacy**	**Disease Model**	**References**
Small molecules	Dihydroartemisin	Reduces inflammation	Rat chondrocytes	Jiang et al., 2016
	Glucocorticoids	Reduces autophagy	Human cartilage	Shen et al., 2015a
	Glucosamine	Reduces OA severity	Nucleus pulposus	Jiang et al., 2014
	Hydroxytirosol	Reduces inflammation	Human chondrocytes	Zhi et al., 2018
		Reduces oxidative stress and apoptosis	OA human chondrocytes and TC28a2 cell line	Cetrullo et al., 2016
	PI3K/AKT/mTOR	Reduces inflammation	Experimental OA in rat	Xue et al., 2017
	Rapamycin	Reduces OA severity	Meniscus mouse OA model	Meckes et al., 2017
		Reduces AGEs	Rat chondrocytes	Huang et al., 2017
		Reduces OA severity and inflammation	Experimental OA mouse model	Ribeiro et al., 2016b
		Reduces catabolic effects	OA human chondrocytes and TC28a2 cell line	Ribeiro et al., 2016a
		Reduces mitochondrial dysfunction and apoptosis	OA human chondrocytes and TC28a2 cell line	Lopez de Figueroa et al., 2015
		Reduces OA severity	Experimental OA mouse model	Takayama et al., 2014
		Reduces OA severity	Experimental OA mouse model	Matsuzaki et al., 2014a
		Reduces mechanical injury damage	Human chondrocytes	Carames et al., 2013
		Reduces OA severity	Experimental OA mouse model	Carames et al., 2012a
	Resveratrol	Reduces OA severity	Human chondrocytes	Qin et al., 2017
	Sucrose	Reduces cartilage degradation/chondrocyte death	Human chondrocytes	Khan et al., 2017
	Trehalose	Reduces oxidative stress	Human chondrocytes	Tang et al., 2017
	Torin 1	Reduces OA severity	Experimental OA mouse model	Chen et al., 2016
		Reduces OA severity	SW1353 cell line and experimental OA rat model	Cheng et al., 2016b
Biologicals	Parathyroid hormone (1-34)	Reduces OA severity	Experimental OA rat model	Chen et al., 2018
	Globular adiponectin	Reduces OA severity	Rat chondrocytes	Hu et al., 2017
	Platelet rich plasma	Chondroprotection/reduces inflammation	Human cartilage	Jiang et al., 2016
	Adipose-stem cells	Reduces catabolic effect/inflammation	Rat chondrocytes	Zhang et al., 2018
Gene Therapy	miR-20	Cell proliferation and autophagy	Chondrocytes	He and Cheng, 2018
	miR-30b	Reduces apoptosis and cartilage degradation	ATDC5 cell line	Chen et al., 2016
	Nanoparticules based siRNA (NFkb)	Reduces inflammation and apoptosis	Experimental OA mouse model	Yan et al., 2016

Preclinical studies targeting autophagy and related molecular mechanisms in cartilage with advanced therapies medical products such as gene and cell therapy have also received some attention. Intra-articular treatment with Parathyroid hormone (1–34) alleviates cartilage degeneration by activating autophagy (Chen et al., 2018). Adiponectin protects chondrocytes from apoptosis related to oxidative stress by inducing autophagy possibly via PI3K/Akt/mTOR (Hu et al., 2017). Other biological products such as Platelet rich plasma (PRP), adipose-stem cells, and serum have also shown efficacy at protecting from degeneration and inflammation through autophagy-related mechanisms in cartilage disease (Jiang et al., 2016; Moussa et al., 2017; Zhang et al., 2018).

Gene therapy and genome editing by nucleases has dramatically expanded the toolbox for the identification and therapeutic validation of targets. Agents interfering with transcription to achieve therapeutic benefit have also received special attention. Nanoparticles targeting NF-κB not only are bioavailable, but reduce early apoptosis, synovitis, and maintain cartilage homeostasis by enhancing AMPK signaling that ultimately attenuate inflammation (Yan et al., 2016). Other examples include miRNA targets (miR-20, miR-30b) that maintain cartilage homeostasis by enhancing autophagy in chondrocytes (Chen et al., 2016; He and Cheng, 2018). One would expect a dramatic transformation of drug discovery pipelines by the generation of engineered cell-based models with disease-relevant genetic modifications in the upcoming years.

### Current and future therapeutic approaches of inflammation in OA

OA is now considered an inflammation-associated multifactorial disorder. As compared to rheumatoid arthritis, the inflammation in OA is chronic, comparatively low grade and mainly mediated by the innate immune pathways including complement and pattern-recognition receptors such as damage associated molecular patterns (Robinson et al., 2016). The recent identification of novel regulators (Liu-Bryan and Terkeltaub, 2015) of inflammation in OA holds promise for the development of transformative therapies. Among these emerging regulators, the bioenergy sensors serine/threonine kinase AMPK and sirtuin-1 (SIRT1) have received particular attention. The role of AMPK and SIRT1 is actually not restricted to energy metabolism, but includes resistance to stress and inflammation. It is noteworthy their role in the tuning of mitochondrial activity to adapt energy consumption, in the resolving of inflammation via the down-regulation of NF-κB, in the control of matrix catabolic responses and last but not least in promoting autophagy. These wide range activities of AMPK and SIRT1 make it a promising entry point for the development of novel therapeutics. A large variety of AMPK activators such as aspirin (Hui et al., 2016), metformin or methotrexate are clinically used in inflammatory disorders (O'Neill and Hardie, 2013). Their use in OA is conceptually attractive and sound, and the results of randomized placebo-controlled trials will certainly bring conclusive data.

The positioning of such sensors at the crossroad of inflammation and autophagy paves the way of research avenues to identify the missing links between chronic inflammation and metabolism alterations related to aging, exercise, nutrition or even circadian rhythm (CR) (Berenbaum and Meng, 2016). CR not only interferes with several physiological processes ranging from behavioral and physiological activities such as sleep/wake cycles, blood pressure, body temperature and metabolism, but also contributes to the severity of aging-related disorders (Longo and Panda, 2016). CR is centrally regulated by a core pacemaker located in the hypothalamus and by an integrated network of peripheral oscillators in tissues and cell types. The coordinated activity of this network controls diurnal pattern of rest and activity in mammals. The pacemaker consists in a group of genes coding for transcriptional activators/repressors that interact in a 24 hourly-tuned feedback loop. The rhythmic activity of the positive regulators brain and muscle aryl hydrocarbon receptor nuclear translocator-like protein 1 (BMAL1) and circadian locomotor output cycles kaput (CLOCK) drives the expression of E-box promoter containing genes that are pivotal in cell differentiation. Among these clock-controlled genes, the negative regulators cryptochrome (CRY) and mammalian period (PER) in turn repress the expression of BMAL1 and CLOCK, thereby completing the feedback loop that cycles every 24 h. While it remained poorly understood, pain and stiffness in OA patients intriguingly follows a daily pattern notably for hand, knee and hip OA. On the other hand, it has been recently identified a circadian clock in chondrocytes (Gossan et al., 2013) that controls the expression of genes involved in cartilage homeostasis and can be altered during aging. Together these data provide a rationale for questioning the role of CR alteration in OA. Following the pioneering description of BMAL1/CLOCK in chondrocytes, it has been subsequently demonstrated that inflammatory cytokines such as IL-1β, conversely to TNF-alpha, severely affects the expression of BMAL1/CLOCK through functional interference with NF-kB (Guo et al., 2017). More recently, it has also been demonstrated that BMAL1, which is essential to maintain the differentiated phenotype of chondrocytes through its interaction with a large number of TGF-β signaling members (Akagi et al., 2017), was repressed in OA and associated with a switch of catabolic phenotype (Snelling et al., 2016). These converging reports have culminated in the description that genetic ablation of BMAL-1 specifically in mouse chondrocytes deeply affects intrinsic CR, contribute to induce a catabolic profile in chondrocytes and leads to cartilage damage (Dudek et al., 2016). Interestingly, it has also been described that BMAL1 predisposes to a marked degeneration of the fibrocartilaginous intervertebral disc (Dudek et al., 2017). Altogether, these data support the notion that disruption of chondrocyte CR may predispose individuals to the onset of cartilage disorders. In search of novel therapeutic interventions in OA, it seems thus reasonable to speculate that the development of drugs interfering with the peripheral clock in cartilaginous tissues may open new therapeutic spaces in the management of OA.

Consistent with this idea, the recent description of a molecular dialog between clock gene BMAL1 and the energy/nutrient sensor SIRT1 has shed further light on the clinical translatability of this therapeutic concept (Dvir-Ginzberg et al., 2016). SIRT1 is well-known to form a regulatory complex with CLOCK/BMAL1 that represses clock gene expression notably in muscle and heart. Such a crosstalk has also been described in human OA cartilage (Yang et al., 2016), where a direct SIRT1-mediated circadian regulation of BMAL1 occurs. Strikingly and of particular clinical applicability, the sirtuin-activating molecule resveratrol, also known as an interacting agent with the aryl hydrocarbon receptor, is well-known to exert beneficial effects on cartilage anabolism. To address the clinical efficacy of such a molecule, the effects of oral resveratrol are currently being assessed in a phase 3 double blind placebo-controlled and randomized clinical trial (NCT02905799-Arthrol) in OA patients.

Also in line with the concept that cartilage intrinsic CR may be a pragmatic entry point for the development of integrative therapeutics in OA, is the intriguing connection between circadian genes and autophagy. As extensively described above, autophagy is a key process in the onset of OA and is thus considered a promising therapeutic target. Of interest, BMAL1 has recently been shown to positively regulate autophagy through mTOR in cardiomyocytes (Qiao et al., 2017). While it remains to be determined whether such a relationship also operates in cartilage, it raises the possibility that restoring the cartilage CR may contribute to increase autophagy processes which in turn contribute to limit cell stress, inflammation and catabolism. Among the molecules that could interfere with central and peripheral CR, melatonin has been widely studied in the context of rheumatologic disorders (Jahanban-Esfahlan et al., 2017). Melatonin is a circadian multi-tasking hormone mainly produced in the pineal gland but also in a wide range of other tissues. Melatonin exhibits diverse biological activities such as anti-oxidation, anti-inflammation or anti-apoptosis through its interaction with specific plasma membrane or nuclear receptors and intracellular targets. In the context of OA, intraarticular injection of melatonin protected against cartilage damages induced by partial meniscectomy in rabbits (Lim et al., 2012). Deciphering the underlying mechanisms has revealed that the anti-inflammatory effect of melatonin could be mediated by a SIRT1-dependant inhibition of the NF-kB pathway (Lim et al., 2012). Consistent with a role of cartilage clock in OA, low doses of oral melatonin, in association with exercise, was found to concomitantly restore the OA-associated reduced levels of clock-controlled genes and reduce the severity of cartilage damages in a murine model of collagenase-induced OA (Hong et al., 2017). This converging body of evidence suggesting that melatonin exerts a protective effect in OA remains however to be consolidated through well-conducted *in vitro* and *in vivo* experiments in animal model of OA or in clinical trial, notably with respect to the ambiguous role of SIRT1 in mediating the pro-inflammatory effects of IL-1β or H_2_O_2_ in chondrocytes (Guo et al., 2017; Hong et al., 2017).

**Figure 1:**
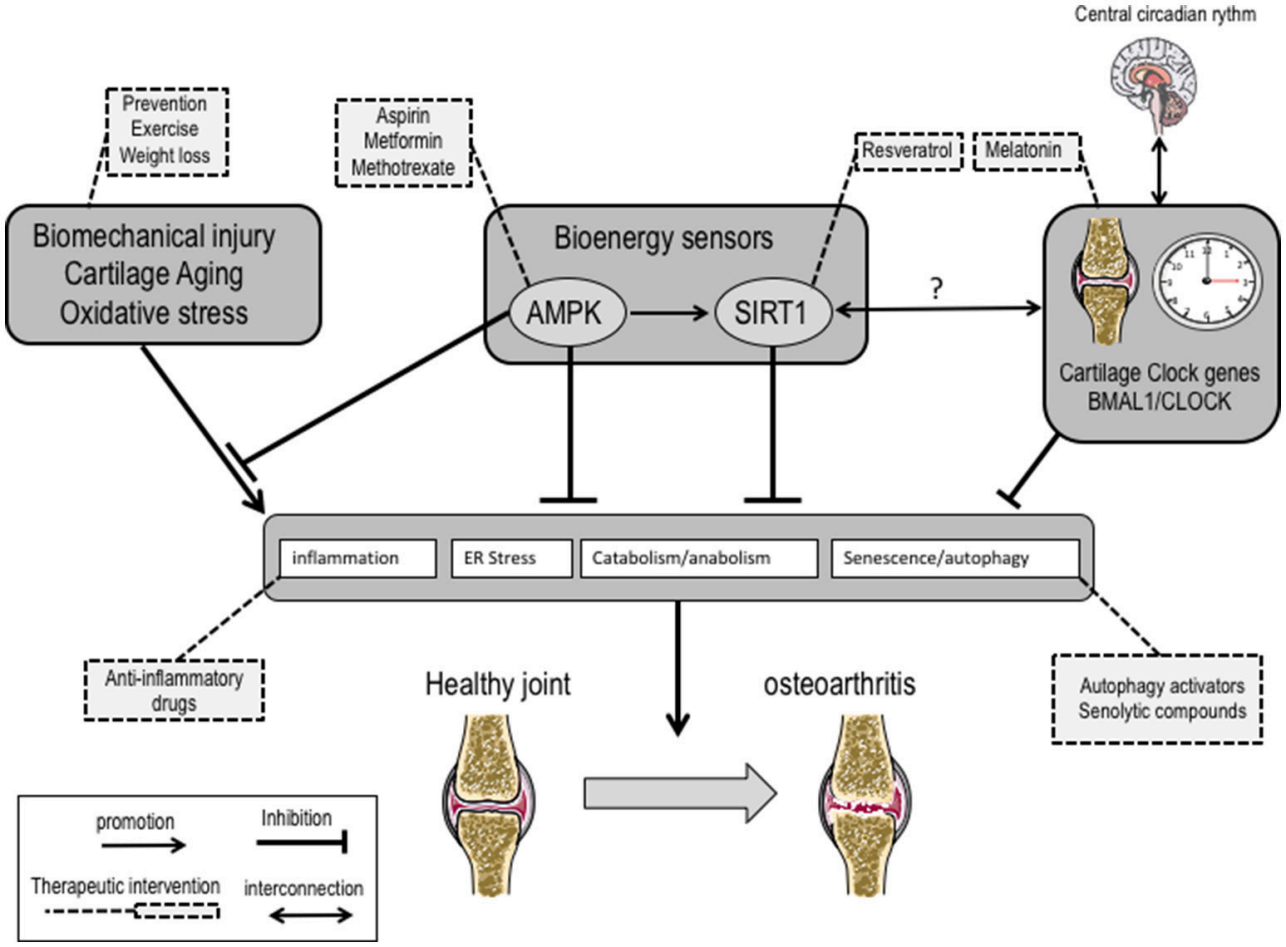
Hypothetical mechanisms underlying the putative role of the circadian rhythm and energy sensors in the inflammation-associated phenotype switch of chondrocytes in osteoarthritis: from molecular actors to therapeutic potential interventions.

Despite some areas of darkness still subsist notably regarding the molecular links between CR, SIRT1/AMPK energy sensors, and autophagy, these data reinforce the notion that local clock genes may be considered as clinically relevant targets for the development of novel therapeutic intervention. See Figure 1 summarizing hypothetical mechanisms underlying the role of the circadian rhythm in OA.

## Conclusions

**Figure 2:**
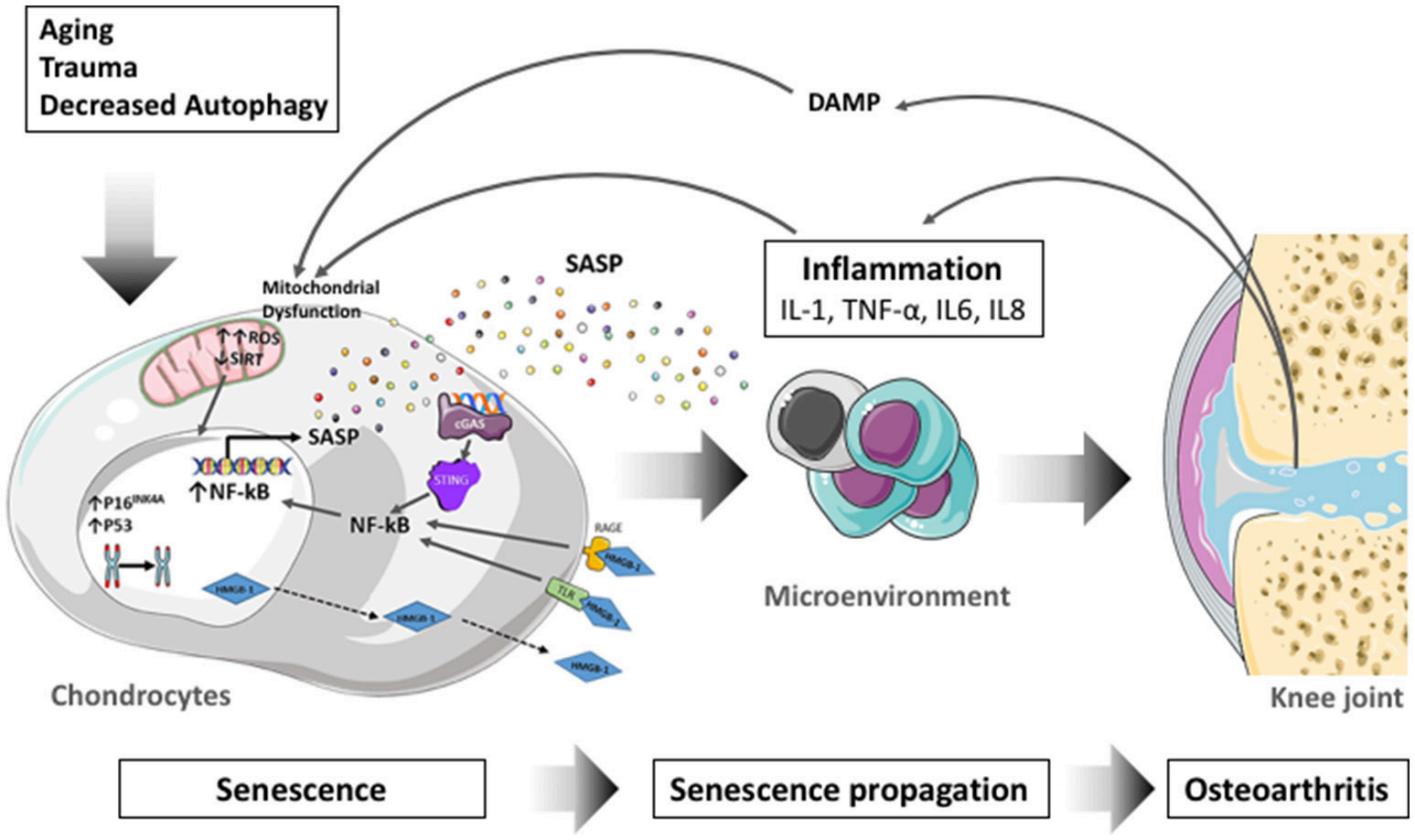
Schematic diagram illustrating the interrelation between autophagy, senescence and inflammation in the onset of osteoarthritis (OA). During aging, after trauma or stress associated with to decreased autophagy and increased oxidative stress, chondrocytes become senescent and secrete a Senescence-Associated Secretory Phenotype (SASP). Factors secreted in the SASP propagate senescence and inflammation to surrounding cells and tissues through a paracrine process and participate to OA. Moreover, the cartilage degradation products called damage associated molecular patterns (DAMP) reinforce inflammation and senescence notably via an increase in oxidative stress (ROS). ROS, Reactive oxygen Species; SIRT, Sirtuin; SASP, senescence associated secretory phenotype; NF-kB, nuclear factor-kappa B; cGAS, Cyclic GMP-AMP synthase; STING, stimulator of interferon genes; TLR, Toll-like Receptor; RAGE, Receptor for Advanced-Glycation End Products; HMGB-1, High Mobility group Box-1; IL-1, interleukin-1; TNF, tumor necrosis factor; IL6, interleukin 6; IL8, interleukin 8; DAMP, Damage-Associated molecular pattern.

Failures of autophagy homeostasis lead to cell dysfunction senescence and death and initiate the musculoskeletal degeneration and inflammation characteristic of rheumatic diseases. In this sense, preclinical models of such fundamental homeostasis mechanisms might create opportunities to build biobanks with patient derived material that can be used to perform drug screens and facilitate drug development. Clinical and preclinical models include blood, tissues from human joints and experiments in rodents showing that aging and OA of articular cartilage are invariably associated with defective autophagy and inflammation. Studying these dimensions of cartilage aging, chondrocyte senescence, inflammation and autophagy could facilitate to open the pharmacological space to novel disease-modifying therapies for OA. In this review, we highlighted what is currently known about preclinical models of OA homeostasis, the major targets and pathways identified, and its potential value for translation. See Figure 2 illustrating the interrelation between autophagy, senescence and inflammation in OA.

## Review criteria

Data for this review were collected by searching in PubMed database for articles published from 1965 to 2018 using the following keywords “osteoarthritis,” “inflammation,” “aging,” “senescence,” “SASP,” “chondrocyte,” “homeostasis,” “autophagy,” “therapeutics,” “metabolism,” “circadian clock” alone or in combination. English-language original publications and Review articles were selected on the basis of their relevance for the inclusion in the bibliography.

## Author contributions

All authors approved the final version to be published. CV, ED, JG, BC: manuscript design and drafting and revising the manuscript.

### Conflict of interest statement

The authors declare that the research was conducted in the absence of any commercial or financial relationships that could be construed as a potential conflict of interest. The handling Editor declared a past co-authorship with one of the authors BC.
